# Neuroprotective Effects and Mechanisms of Senegenin, an Effective Compound Originated From the Roots of Polygala Tenuifolia

**DOI:** 10.3389/fphar.2022.937333

**Published:** 2022-07-18

**Authors:** Zhe Chen, Yu Yang, Ying Han, Xijun Wang

**Affiliations:** National Chinmedomics Research Center, National TCM Key Laboratory of Serum Pharmacochemistry, Metabolomics Laboratory, Department of Pharmaceutical Analysis, Heilongjiang University of Chinese Medicine, Harbin, China

**Keywords:** senegenin, *Polygala tenuifolia* Willd., biological activity, neuroprotective effect, Chinese medicine

## Abstract

Senegenin is the main bioactive ingredient isolated from the dried roots of *Polygala tenuifolia* Willd. In recent years, senegenin has been proved to possess a variety of pharmacological activities, such as anti-oxidation, anti-inflammation, anti-apoptosis, enhancement of cognitive function. Besides, it has a good development prospect for the treatment of neurodegenerative diseases, depression, osteoporosis, cognitive dysfunction, ischemia-reperfusion injury and other diseases. However, there is no systematic literature that fully demonstrates the pharmacological effects of senegenin. In order to meet the needs of new drug research and precise medication, this review summarized the neuroprotective effects, mechanisms and gastrointestinal toxicity of senegenin based on the literatures published from the past 2 decades. In addition, an in-depth analysis of the existing problems in the current research as well as the future research directions have been conducted in order to provide a basis for the clinical application of this important plant extract.

## 1 Introduction

Polygalae Radix (Yuanzhi), the dried root of *Polygala tenuifolia* Willd., is mainly used to treat insomnia and forgetfulness, palpitations, expectoration, depression (Lin et al., 2012; Choi et al., 2020). So far, multiple chemical components have been discovered in Polygalae Radix (Ling et al., 2013), among which Polygalae Radix saponins are the active ingredient and the main toxic component of Polygalae Radix (Guan et al., 2012). However, due to the lack of toxicity studies on Polygalae Radix saponins, scholars have not documented any side effects other than gastrointestinal irritation with Polygalae Radix, the application and development of its new products are limited.

Undoubtedly, senegenin (13-(chloromethyl)-2,3-dihydroxy-4,6a,11,11,14b-pentamethyl-2,3,4,4a,5,6,6a,7,8,9,10,11,12,12a,13,14,14a,14b-octadecahydropicene-4,8a (1H)-dicarboxylic acid) is one of the most important active ingredients in Polygalae Radix. Firstly, it is noteworthy that saponins from Polygalae Radix can be converted into senegenin by hydrolysis and metabolism. And experiments have shown that after removing the glycosyl and turning Polygalae Radix saponins into senegenin, the toxicity can be reduced (Wen et al., 2015). Secondly, due to the lipophilicity and small molecular size (Mw 537), senegenin can easily pass through the blood-brain barrier to exert pharmacological effects. It has a pronounced effect on enhancing cognitive function, improving intelligence and preventing injury (Chen et al., 2010; Lv et al., 2016; Pi et al., 2016).

**FIGURE 1:**
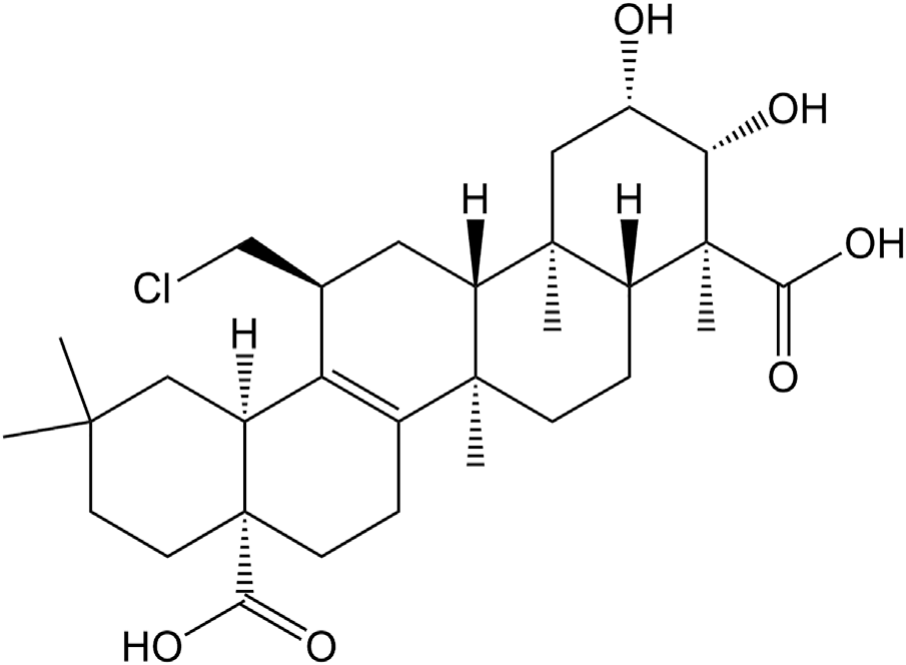
The molecular structure of senegenin.

**FIGURE 2:**
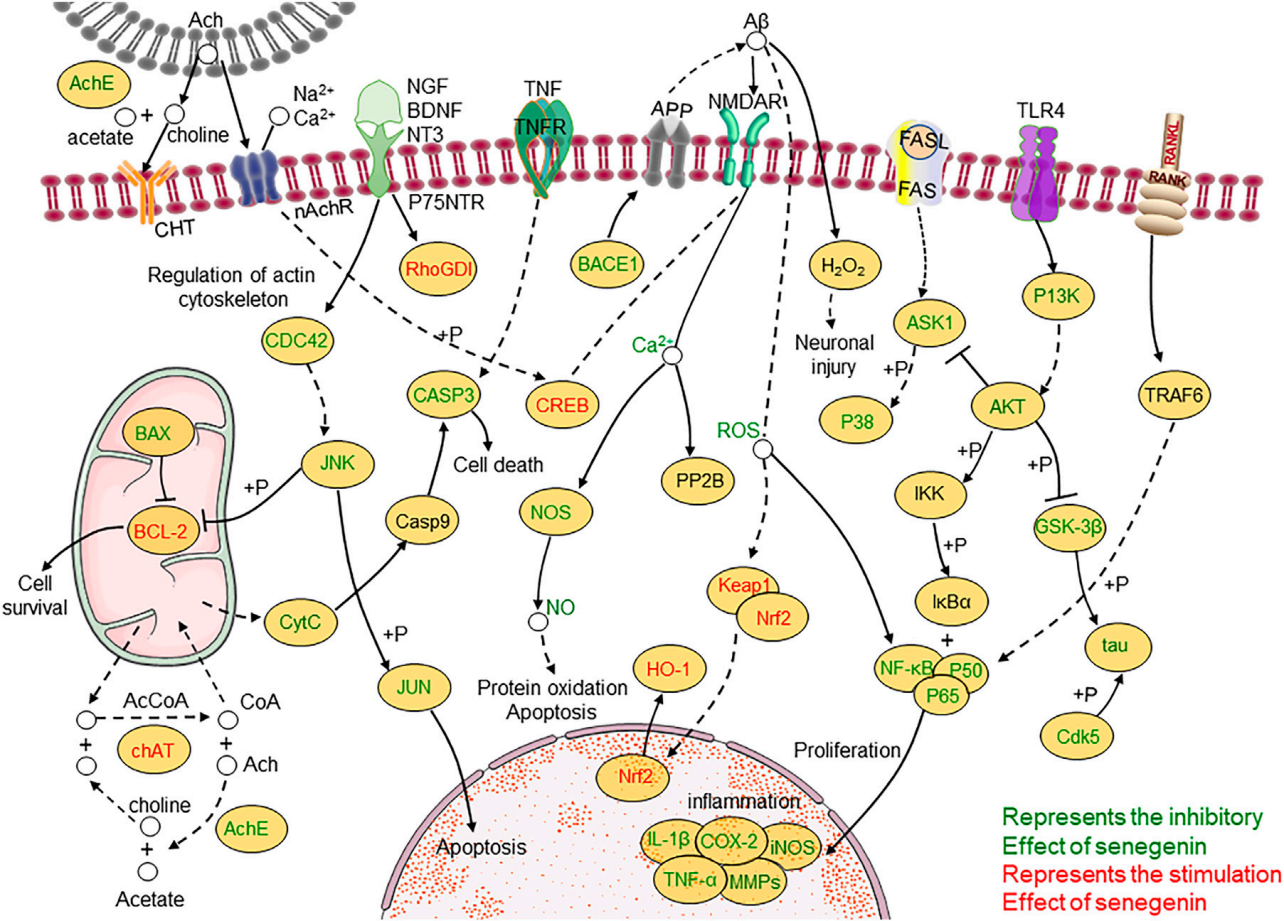
Molecular pathways involved in the pharmacological activities of senegenin.

At the same time, clinical studies have also found that after adding *β*-asarone and senegenin to memantine, the treatment of moderate-to-severe AD was significantly enhanced, with good acceptability and no other side effects (Chang and Teng, 2018; Dong et al., 2018). There is potential for senegenin as a clinical therapy in the future, and its pharmacological research has high value for precise treatment and clinical application. This paper provides a systematic review of senegenin, and lays the foundation for follow-up clinical research and application of senegenin. The molecular structure of senegenin is shown in Figure 1. The specific molecular pathways are shown in Table 1 and Figure 2.

**TABLE 1:** Molecular mechanisms to the pharmacological activity of senegenin.

References	Models	Type and administration route	Effective doses	Molecular mechanisms
**Anti-neurodegenerative diseases**				
Jesky and Chen. (2016)	Aβ_25–35_-induced PC 12 cells	*In vitro*	40 and 20 μg/ml	Up-regulation of MAP2 and Gap-43
Zhang et al. (2011)	Rat hippocampal neurons	*In vitro*	20 and 40 μmol/L	Up-regulation of SOD and Bcl-2, down-regulating the level of MDA, Bax and caspase-3
Liang et al. (2011)	Human neuronal 6-OHDA-injured SH-SY5Y cells	*In vitro*	1.0 × 10⁻^1^–10 μM	Increasing SOD, GSH, MMP levels, inhibiting caspase-3 activation, and stimulating TH expression
Sun et al. (2007)	H_2_O_2_-induced Injury of PC12 cells	*In vitro*	20 and 10 mg/L	Improving the activity of SOD, Decreasing the level of MDA
Chen et al. (2015)	Aβ_1-40_-induced AD rats	*In vivo;* oral administration	37.0 and 74.0 mg/kg, 30 days	Down-regulating ub expression, up-regulating the activity of ubiquitin ligase E3 and 26S proteasome by regulating UPP pathway
Pi et al. (2016)	Primary cortical neurons from Neonatal SD rats	*In vitro*	2 µM	Activating PI3K/Akt pathway
Chen et al. (2012a)	Aβ_1-42_-induced AD rats	*In vivo*; oral administration	500 mg/(kg/d),750 mg/ (kg/d),1,000 mg/(kg/d), 30 days	Decreasing the level of GSK-3β, CDK-5, increasing PP-1 and PP-2A
Xu and Chen. (2012)	Aβ_1-40_-induced AD rats	*In vivo*; oral administration	18.5, 37.0, and 74.0 mg/kg, 30 days	Increasing PP-2A, down-regulating PKA, and inhibiting hyperphos-phorylation of the MAPT Ser396 in rat brain neurons
Lv et al. (2016)	LPS-induced RAW 264.7 cells	*In vitro*	0.98, 1.86, 3.72 and 7.44 μM	Decreasing the expression of iNOS and COX-2, up-regulating HO-1 by inhibiting MAPK/NF-kB and activating the KEAP-Nrf2-ARE pathways
Huang et al. (2018)	STZ-induced AD rats	*In vivo*; oral administration	2, 4, and 8 mg/kg, 28 days	Increasing the SOD and GSH-Px activities, inhibiting the phosphorylation of tau proteins
Yang et al. (2013)	Aβ_1-40_-induced PC12 cells	*In vitro*	50,100 and 200 μmol·L-1	Inhibiting the expression of Bax , Cyt c and increasing Bcl- 2
Wang et al. (2017)	LPS- induced Murine BV2 microglia cells and Male ICR mice	*in vitro and in vivo*; oral administration	1–4 μM; 5, 10, 20 mg/kg, 3 weeks	Activation of KEAP-Nrf2-ARE level and Nrf2-HO-1 pathway
Yuan et al. (2012)	LPS-induced PD rats	*In vivo*; oral administration	200 or 300 mg/kg, 14 weeks	Enhancing the survival rate of TH-ir, improving the level of DA, inhibiting microglial activation and TNF-α, IL-1β
Ye and Chen. (2013)	Aβ_1-40_ induced AD rats	*In vivo*; oral administration	18.5,37.0,74.0 mg/kg, 30 days	Decreasing the levels of Bax, Cyt c, caspase-3 and increasing the expression of Bcl- 2
Jia et al. (2004)	neuroblastoma (SH-SY5Y) cells	*In vitro*	2 ,4 μg/ml	Inhibiting the secretion of Aβ *via* BACE1
Chen et al. (2012b)	neural stem cells	*In vitro*	1, 2, and 4 μg/ml	Increasing BrdU, Tuj-1 and GFAP positive cells
Chen et al. (2006)	Aβ_1-40_ and IBO- induced AD rats	*In vivo*; oral administration	74.0 mg/kg/d, 30 days	Increasing ChAT activity, and decreasing AchE activity
Tian et al. (2004)	SAM-P/8 mice	*In vivo*; oral administration	50 mg/kg, 30 days	Decreasing the AchE and IL-2
Huang et al. (2013)	TPED-induced KM and ICR mice	*In vivo*; oral administration	4 mg/kg, 30 days	Decreasing AChE activity and MDA level, increasing SOD activity
Tian et al. (2022)	Aβ_1-42_ induced mice hippocampal neuron HT22 cells	*In vitro*	10, 20, 40 and 60 μM, 24 h	Recovering MMP, reducing ROS, decreasing p62, promoting PINK1
Ren et al. (2022)	Aβ_1-42_ induced Rat PC12 cells	*In vitro*	0, 10, 30, 60 or 80 μM	Activation of the PI3K/Akt, up-regulating Bcl-2/Bax, enhancing HO-1, promoting Nrf2 nuclear translocation and reducing ROS
Lu et al. (2017)	LPS- induced rat microglia cells	*In vitro*	10^−4^M, 10^−5^M, and 10^−6^M	Decreasing the secretion of NO, MMP-9, TNF-α/IL-1β
**Aniti-POCD**				
Cai et al. (2013)	The OVX mice	*In vivo*; oral administration	4 mg/kg, 30 days	Reducing the loss of NOS positive neurons
Lin et al. (2018)	propofol anaesthesia in maternal rats	*In vivo*; intraperitoneal injection	15 mg/kg	Decreasing HDAC2 and HGN, increasing the acetylation of H3K14, H4K12 and phosphorylation of CREB, NR2B
Xie et al. (2012)	HIR-induced POCD rats	*In vivo*; oral administration	15, 30, and 60 mg/kg, 7 days	Increasing the expression of hippocampal NR2B(NMDA)
Chen et al. (2010)	Primary cultures of rat hippocampal neurons	*In vitro*	1, 2, and 4 μg/ml	Inhibiting caspase-3, reversing down-regulation of the ratio of Bcl-2/Bax
**Neuroprotective effects of ischemia reperfusion**				
Li et al. (2015)	PC12 cells; primary cortical neurons	*In vitro*	15, 30, 60, 70, 80 μM	Up-regulating RhoGDIα expression and inhibiting the phosphorylation of jnk, c-jun, reducing the ratio of Bax/Bcl-2
Zhu et al. (2016)	H/R-induced injury in PC12 cells	*In vitro*	15, 30, 60 and 80 μmol/L	Reducing the accumulation of ROS and the increment of (Ca2+]i by inhibiting the activity of NOX
**Antidepressant effects**				
Li et al. (2017)	CUMS-induced mice depression	*In vivo;* oral administration	4 and 8 mg/kg, 7 days	Inhibition of NF-κB, regulating NLRP3 signal pathway
**Anti-optic nerve injury**				
Bie et al. (2012)	H_2_O_2_-induced RGCs	*In vitro*	10, 20,and 40 μmol/L	Promoting Bcl-2 protein, Down-regulation of caspase-3, Cyt c
**Protective effects of nerve cells in spinal cord**				
Zhang et al. (2016)	Spinal cord contusion-injured rats	*In vivo;* tail vein injection	30 mg/g, 3 days	Decreasing the level of Bax and Caspase-3, increasing the level of Bcl-2
**Other pharmacological effects**				
Yang et al. (2015)	BMM; RANKL-induced bone loss C57BL/6 mice	*In vitro and in vivo*; intraperitoneal injection	8 mg/ml, 3 mg/kg; 3 days	Inhibiting NF-kB pathway
Wang et al. (2016)	IL-1β-induced Primary chondrocytes	*In vitro*	2, 4, 8 μg/ml	Suppressing IL-1β-induced NO, PGE2, MMP-1, MMP-3, MMP-13 and PI3K/AKT/NF-κB pathway
Jia et al. (2017)	LPS/GalN-induced acute hepatic injury in mice	*In vivo;* intraperitoneal injection	2, 4 and 8 mg/kg	Inhibiting the level of NF-κB, MAPKs, ALT, AST, the activities of MDA, MPO, and the expression of ASK1. Activating Nrf2/HO-1 pathway
Fu et al. (2016)	LPS-induced AKI mice	*In vivo;* intraperitoneal injection	2, 4 and 8 mg/kg	Suppressing TNF-α, IL-1βand IL-6, inhibiting TLR4/NF-κB pathway
Xu et al. (2017)	cecal ligation and puncture induced KM mice	*In vivo;* intraperitoneal injection	50, 20 or 5 mg/kg	Suppressing TNF-α, IL-1βand IL-6, inhibiting NO and PGE2, inhibiting NF-κB pathway
Lv et al. (2015)	LPS-induced ALI BALB/c mice and the RAW 264.7 cell	*In vitro and in vivo;* intraperitoneal injection	2, 4 and 8 mg/kg	Inhibition of p38/ERK, IκBα phosphorylation and NF-κBMAPK pathways, decreasing the TNF-α, IL-1β, IL-6, COX-2, reducing the activity of MPO

## 2 Pharmacological Activities

### 2.1 Neuroprotective Effects

Due to aging and a variety of social factors, the incidence of neurological diseases has increased dramatically worldwide (Bertram and Tanzi, 2005; Qiu et al., 2009). It is worth noting that diseases, such as neurodegenerative diseases, cognitive disorders and depression, seriously affect the quality of life of patients and impose a huge burden on families and society.

A variety of factors can cause nerve damage. The increase of intracellular calcium can cause the activation of free radicals and lead to the death of nerve cells through the process of apoptosis. Dysfunction of choline can lead to neurodegenerative diseases (Huerta and Lisman, 1993). Oxidative stress can cause various destructive effects, such as lipid peroxidation, DNA damage and cell death by producing reactive oxygen species (ROS) at high levels (Sugawara and Chan, 2003). While senegenin can eliminate abnormal proteins by regulating UPP (ubiquitin-proteasome pathway), and it can nourish nerve cells, reduce oxidative stress, inhibit inflammatory response, evaluate learning and memory ability and play neuroprotective effects against apoptosis by regulating MAPK/NF-κB, Nrf2/HO-1, PI3K/Akt, ROS/ Ca^2+^ and other pathways.

#### 2.1.1 Anti-Neurodegenerative Diseases

Taking AD as an example, which is a kind of progressive, serious and lethal complex disease, characterized by the loss of a large number of specific neurons. To treat this disease, drugs such as NMDA receptor antagonist memantine and cholinesterase inhibitor donepezil have been used in clinical settings (Piemontese, 2017). Although these drugs can alleviate AD-related symptoms, adverse reactions such as liver toxicity, diarrhea, and vomiting may also occur (Ballard et al., 2011). Therefore, the choice of effective and low toxic drugs will be a potentially effective method.

Natural products play a significant role in the treatment of human disease, which are incomparable source of novel drug leads and inspiration for the synthesis of non-natural drugs. Senegenin, as an important natural product, which can treat neurodegenerative diseases in a variety of ways, and some potential signal transduction pathways have been elucidated.

##### 2.1.1.1 Ubiquitin-Proteasome Pathway

In 1987, scholars proved for the first time that AD is related to abnormal UPP function (Perry et al., 1987). There are a large number of misfolded proteins in brain cells, which are also the main culprits of AD and PD. In order to prevent the accumulation of ineffective proteins from causing cell damage or death, more than 85% of the proteins in eukaryotic cells are mainly degraded through the UPP (ubiquitin-proteasome pathway) (Golab et al., 2004).The degree of UPP dysfunction is significantly correlated with the accumulation of highly phosphorylated microtubule-associated protein tau (Tau) and Aβ in the AD brain (Song and Jung, 2004).

In an *in vitro* experiment, it was found that senegenin can protect PC12 cells from Aβ_25-35_-induced cytotoxicity by increasing the number of protrusions, the average length and maximum length, and the expression of Map2 and Gap-43 (Jesky and Chen, 2016). And after continuous administration of senegenin 37.0 and 74.0 mg/kg to AD rats for 30 days, the expression of Ub was down-regulated, the activity of ubiquitin ligase E3 and 26S proteasome was up-regulated, the aggregation of Aβ_1-40_ was inhibited, the phosphorylation level of Tau Ser396 in hippocampus neurons Aβ_1-40_, hippocampal, and cortical neurons was reduced (*p* < 0.05, *p* < 0.01).

These results indicate that senegenin can enhance the ability of Ub to recognize and bind abnormally accumulated proteins, increase E3 expression and proteasome activity, thereby clearing the abnormal deposition of Aβ and Tau in the brain of AD rats, protecting the structural and functional integrity of neurons, and restoring the vitality of nerve cells. These phenomena suggested that senegenin might be a potential anti-dementia drug.

##### 2.1.1.2 GSK-3β\CDK-5\PKA

Neurofibrillary tangles (NFTs) are the main pathological changes of AD. NFTs are mainly composed of pairhelical filaments (PHF) and dystrophic axons. And PHF mainly contain highly phosphorylated tau protein (Morishima-Kawashima et al., 1995). Among them, the enzymes with the highest phosphorylation efficiency of tau are GSK-3β, CDK-5, PP-1, and PP-2A (Martin et al., 2013), suggesting that neurodegenerative diseases can be controlled by changing the expres-sion of these enzymes.

In an Aβ_1-42_-induced AD experiment, it was found that senegenin can reduce the expression of GSK-3β and CDK-5, up-regulate the activity of PP-1 and PP-2A, and in-hibit the abnormal phosphorylation of tau (Chen Y. et al., 2012). Another study showed that senegenin protected neurons from Aβ_1-40_-induced tau phosphorylation by down-regulating the expression of PKA protein and inhibiting the hyperphosphorylation of MAPT Ser396 (Xu and Chen, 2012). The above experiments prove that senegenin plays a certain therapeutic effect on neurodegenerative diseases by inhibiting the abnormal phosphorylation of tau.

##### 2.1.1.3 NLRP3/Nrf2/HO-1

Once stimulated by infection or injury, Aβ will induce microglia to release inflammatory cytokines (Lau et al., 2007), which may lead to neuronal damage, degeneration and death, and give rise to neurodegenerative diseases (Wyss-Coray and Mucke, 2002). Nuclear factor E2-related factor 2 (Nrf2) has been reported to play an important role in the regulation of many detoxification and antioxidant genes, and cell protection (Ishii et al., 2000). Heme oxygenase (HO-1) is an antioxidant enzyme that participates in the process of heme degradation (Wang et al., 2017). To reduce oxidative stress and inflammation, it is important to regulate the release of Nrf2 and HO-1 (Li et al., 2020).

By activating the Keap-Nrf2-ARE pathway, senegenin can reduce ROS accumulation and up-regulate the expression of Nrf2 and HO-1 in a dose-dependent manner, which contributes to the reduction of oxidative stress (Ren et al., 2022). In addition, senegenin can inhibit the production of PGE_2_ and NO in RAW 264.7 macrophages induced by lipopolysaccharide (LPS) by suppressing MAPK/NF-κB and activating the Nrf2/HO-1 signaling pathway, and reduce inducible nitric oxide synthase (NOS) and Cyclooxygenase-2 (COX-2) gene expression (Lv et al., 2016; Lu et al., 2017).

In addition, senegenin has been reported to increase striatal dopaminergic levels, which enhanced the survival rate of tyrosine hydroxylase-positive neurons in SNPC to 75% at the dosage of 300 mg/kg. It was found that senegenin can reduce the production of reactive oxygen in BV2 microglia, and inhibit the activation of NLRP3 inflammasomes, the division of caspase-1 and the secretion of IL-1β(Yuan et al., 2012), moreover, senegenin inhibits memory loss in mice in a significant way (Fan et al., 2017). It is proved that senegenin can significantly improve the degeneration of dopaminergic neurons and inhibit the activation of NLRP3 inflammasomes in the substantia nigra.

These findings suggested that senegenin has a good prospect of clinical application in neurodegenerative diseases therapy, and the underlying mechanisms were implicated in the inhibition of cell inflammatory response and the improvement of the survival rate of damaged neurons.

##### 2.1.1.4 PI3K/Akt

Excessive nerve cell loss induced by synapse loss and axon damage is a common feature of many brain diseases, especially AD, PD and Huntington’s disease (Christensen et al., 2010). Studies have shown that several endogenous neurotrophic factors (NTFs), brain-derived neurotrophic factor (BDNF), nerve growth factor (NGF) play a key role in neuron survival, neurite outgrowth, synaptic connections, and nervous system plasticity (Mullen et al., 2012). However, as a polypeptide, NTFs are difficult to penetrate the blood-brain barrier, and their metabolism in the body is fast, making it impossible for clinical application (Pardridge, 2002).

The function of senegenin is similar to BDNF and it has been determined to promote the growth of axon protrusions and neuron survival (Pi et al., 2016). Senegenin significantly reduced the LDH content in the culture medium of the senegenin group, and the reduction was dose dependent. Additionally, senegenin increased the expression of MAP2 mRNA and BDNF mRNA in cortical neurons of newborn rats, proving that senegenin has neurotrophic properties (Pi et al., 2013). There are experiments using different pharmacological antagonists to treat primary cultured rat cortical neurons. They found that A2A receptor antagonists and PI3K inhibitors can significantly inhibit senegenin-induced neurite outgrowth and neuron survival. In addition, senegenin can also promote the phosphorylation of Akt (Pi et al., 2016) It reveals that the PI3K/Akt signaling pathway may be involved in the neurotrophic effect of senegenin. In addition, through the PI3K/Akt signaling pathway, senegenin can also increase the ratio of P-PI3K/PI3K and P-Akt/Akt, increase cell survival in a dose-dependent manner, and reduce apoptosis (Ren et al., 2022).

##### 2.1.1.5 Bcl-2/Bax

Reactive oxygen species (ROS) exist in the normal metabolic process of cells in a state of dynamic equilibrium. Oxidative stress may change the number and structure of hippocampal neurons and synapses (Mittler, 2017). As an antioxidant, Bcl-2 plays a special role in buffering the production of mitochondrial reactive oxygen species and delaying the progression of the cell cycle (Agostinis, 2009). In addition, there has been evidence that the apoptosis of AD nerve cells is closely related to the Bcl-2 gene family (Clementi et al., 2006). Senegenin can reduce oxidative stress and inhibit cell apoptosis by regulating Bcl-2/Bax.

A study reported that senegenin had a protective effect on PC12 cells induced by H_2_O_2_ and enhanced the neuronal survival in dose-effect manner (Jang and Surh, 2004; Zhu et al., 2005; Zhang et al., 2011). Furthermore, senegenin can significantly reduce the amount of LDH, and the content of MDA, improve the activity of SOD and the cell survival rate (Sun et al., 2007). Senegenin significantly reduced the damage of cells by recovering cell viability and MMP, reducing apoptosis and ROS. Mitophagy is activated by PINK1/Parkin translocation to mitochondria, which is mediated by senegenin down-regulation of p62, accumulation of full-length PINK1(Tian et al., 2022). Senegenin can increase Bcl-2 protein expression, inhibit Bax protein expression, and increase Bcl-2/Bax ratio, thereby inhibiting the formation of Bax dimers, preventing Cyt c leakage, protecting mitochondrial structure, finally inhibiting PC12 nerve cell apoptosis.

In summary, senegenin can improve the activity of hippocampal neuron antioxidant enzymes, inhibit cell apoptosis to exert protective activity on neurons.

##### 2.1.1.6 Acetylcholinesterase

Learning and memory impairment is the main clinical symptom of AD. For a long time, cholinergic transmission in the hippocampus has been regarded as an important regulator of synaptic plasticity, memory consolidation and other cognitive processes (Huerta and Lisman, 1993).

Experiments have shown that the senegenin-mediated enhancement of learning and memory in the Y maze may be due to the increase of cholinergic synaptic transmission (Huang et al., 2013). 4 mg/kg senegenin can significantly reduce hippocampal acetylcholinesterase (AChE) activity and malondialdehyde (MDA) level, and increase the activity of superoxide dismutase (SOD). In the electrophysiological experiment of hippocampal slices, 2 g/ml senegenin significantly enhanced the basal synaptic transmission and the amplitude of the field excitatory postsynaptic potential (FEPSP) after high-frequency stimulation of Schaffer to CA1 pathway (Scha-CA1). It is suggested that senegenin enhances the learning and memory ability of mice by inhibiting the activity of AChE, improving the antioxidant capacity, and enhancing synaptic plasticity. Therefore, senegenin shows potential prospects in improving learning and memory.

#### 2.1.2 Anti-Postoperative Cognitive Dysfunction

Postoperative cognitive dysfunction (POCD) is a new form of cognitive dysfunction that occurs after surgery. There is evidence that POCD occurs in 41% of surgical patients over 60 years old, and 50% of those patients will suffer permanent dysfunction, which is associated with higher mortality rates (Holečková et al., 2018). Congestive heart failure, hypertension, age, diabetes mellitus, previous stroke/transient ischemic attack, anesthetics and other factors have all been proved to be related to the occurrence of POCD (Medi et al., 2013; Hou et al., 2018).

Despite the fact that the exact cause of POCD is unclear, cholinergic failure in the central nervous system is believed to be its underlying biological basis (Holečková et al., 2018; Kotekar et al., 2018). Senegenin, as a pharmacological drug that inhibits acetylcholinesterase, is expected to play a significant role in the treatment of POCD.

##### 2.1.2.1 NR2B

Hepatic vascular blockade is a common method for liver surgery to treat end-stage liver disease. The resulting Hepatic ischemia reperfusion (HIR) may lead to cell death, systemic inflammation, and multiple organ failure, cognitive dysfunction (Colletti et al., 1996). And NR2B expression defects may play a crucial role in age-related cognitive decline (Hawasli et al., 2007).

An experiment had found that the memory retention ability of HIR rats in the platform jumping experiment and Y maze experiment is impaired, which manifests as hippocampus-dependent memory impairment, and is accompanied by a decrease in the expression of NMDA receptor NR2B subunit mRNA and protein in the hippocampus. The cognitive impairment caused by HIR can be reduced in a dose-dependent and time-dependent manner after intragastric administration of senegenin 60 mg/kg (Xie et al., 2012). It is suggested that senegenin may improve cognitive impairment by increasing NR2B, and increase the protective effect of senegenin on nerve injury of POCD or other neurodegenerative diseases related to NMDA dependent signal pathway dysfunction, such as AD, vascular dementia and age-related cognitive decline.

##### 2.1.2.2 Nitric Oxide Synthase

Researchers have found that the loss of nNOS caused by hypoestrogenism or ovary removal is associated with cognitive and mood disorders (Walf et al., 2009), which may lead to changes in the morphology of hippocampus and prefrontal cortex synapses (Wallace et al., 2006). However, traditional estrogen therapy increases the risk of breast cancer and venous thromboembolism (Stahlberg et al., 2004; Olié et al., 2010), restricting traditional estrogen therapy. Recently, experiments have shown that senegenin can reduce the loss of nNOS and enhance learning and memory ability.

Some scholars use dark avoidance and Y maze experiments to evaluate learning and memory ability and found that 4 mg/kg senegenin can significantly prolong the incubation period of ovariectomized (OVX) mice. The mice in senegenin group reached the learning standard (90% correct) on the 9th day after training (Rao et al., 2001), which suggested that senegenin can improve the learning and cognitive ability (Su et al., 2010; Cai et al., 2013). Moreover, experiments have found that administration of senegenin can significantly reduce the loss of NADPH-d-positive neurons caused by ovariectomy in the hippocampus of mice, and prevent changes in synaptic morphology after ovariectomy (Cai et al., 2013). It is suggested that reducing the loss of NADPH-d positive neurons may be one of the mechanisms of senegenin to improve learning and memory impairment.

All in all, senegenin can improve learning and memory impairment in ovariectomized mice, reduce the loss of nNOS-positive neurons in the hippocampus caused by ovariectomy, and prevent the morphological changes of synaptic ultrastructure, suggesting that senegenin may play a potential therapeutic value in improving neurological symptoms and cognitive dysfunction caused by surgical menopause.

##### 2.1.2.3 HDACs-CREB-NR2B

Propofol is widely used in clinics, including non-obstetric surgery for pregnant women. However, it is reported that propofol affect the learning and memory of offspring by inhibiting histone acetylation in early pregnancy, damage hippocampal neurons and may lead to POCD (Barria and Malinow, 2005).

In an experiment that rats were given propofol in early pregnancy (E7) to simulate the learning and memory dysfunction of young rats, the results showed that senegenin promoted synaptic plasticity by upregulating NR2B in the hippocampus, promoting learning and memory, and down-regulated the expression of Hippyragranin (HGN), a negative regulatory protein that is highly expressed in the hippocampus (Zhang et al., 2005). In addition, histone deacetylase (HDACs) inhibitors can promote synaptic plasticity and memory, and activate CREB-mediated transcription (Vecsey et al., 2007; Fujita et al., 2012; Fass et al., 2013). Senegenin may improve the learning and memory impairment of offspring rats through the HDACs-CREB-NR2B pathway (Luo et al., 2016; Lin et al., 2018). Notably, the BDNF-TrkB signaling pathway is one of the downstream regulatory targets of histone acetylation. Upregulation of HDAC2 has been confirmed to damage the BDNF-TrkB signaling pathway (Ji et al., 2014), suggesting that senegenin also protects learning and memory through BDNF-TrkB pathway.

##### 2.1.2.4 Caspase-3/Bcl-2/Bax

Diabetes mellitus not only causes physical complications, but it may also accelerate cognitive dysfunction and increase the incidence of POCD (Jones, 2008). Bax and Bcl-2 are two important apoptosis regulators in the Bcl-2 family, Bcl-2 can postpone the occurrence of diabetes and cognitive dysfunction (Agostinis, 2009).

Senegenin (1–4 μg/ml) pretreatment can dose-dependently inhibit the production of reactive oxygen species in hippocampal nerve cells induced by methylglyoxal (100 μM) and which can alleviate the cognitive dysfunction caused by diabetes and POCD. The underlying mechanism is related to decreasing the activity of caspase-3 and increasing the ratio of Bcl-2/Bax (Chen et al., 2010). These studies indicated that senegenin has an inhibitory effect on methylacetaldehyde-induced cell death, which can interfere with the execution of the apoptosis program and facilitate the formation of Bcl-2-Bax heterodimers (Simon et al., 2000).

#### 2.1.3 Neuroprotective Effects of Ischemia Reperfusion

There is a high morbidity and mortality rate associated with ischemic cerebrovascular disease. In clinical practice, blood flow reperfusion after cerebral ischemia is usually necessary. Paradoxically, reperfusion after prolonged ischemia can produce harmful effects such as calcium overload, free radical production and mitochondrial changes, which can aggravate neuronal cell damage and even cell death. At present, the pathogenesis of ischemia/reperfusion (I/R) injury is complex and is not yet fully clear, the recognized mechanisms include oxidative stress (Prakash et al., 2011), inflammation (Ma et al., 2013; Yin et al., 2013), intracellular free calcium ion overload (Tang et al., 2011), as well as delayed neuronal death (Shioda et al., 2006). According to several reports, senegenin has anti-apoptotic and anti-oxidant effects in the process of hypoxia/reoxygenation (H/R) injury, moreover, it could protect nerves from damage.

##### 2.1.3.1 RhoGDI/JNK

More and more evidences indicated that cerebral ischemia-reperfusion can activate Rac1/Cdc42 and induce neuronal apoptosis (Raz et al., 2010). The results of proteomics combined with molecular biology techniques have proved the downstream signaling mechanism of the protective effect of senegenin on H/R-induced PC12 cell apoptosis, that is, up-regulating the expression of RhoGDIα, which is the upstream signaling molecule of the Rac1/Cdc42 pathway (Boulter et al., 2010). And the level of RhoGDIα protein was significantly increased (Garcia-Mata et al., 2011), while the level of active Rho protein (Rac1 and Cdc42) was obviously decreased. Furthermore, senegenin can inhibit the phosphorylation of jnk and c-jun, and reduce the ratio of bax/bcl-2 (Li et al., 2015). These data proved that senegenin has certain pharmacological activity against H/R by blocking the apoptosis of nerve cells.

##### 2.1.3.2 Reactive Oxygen Species/Ca^2+^

Son et al. (2010) believed that increasing Ca^2+^ in the cytoplasm could lead to various harmful effects, including weakening cellular antioxidant defense and increasing membrane permeability, which would promote the loss of mitochondrial membrane potential (Ψm), and finally stimulate the generation of ROS and forming a positive feedback loop. However, the NADPH oxidase of the NOX family gene deletion can protect mice lungs from ROS caused by ischemia (Al-Mehdi et al., 1998).

It is well known that damage mediated by H/R can trigger the mitochondrial-dependent apoptosis pathway (Sarkey et al., 2011). At the same time, the loss of *Ψ*m has been shown to be a hallmark of mitochondrial-dependent apoptosis (Skulachev, 2006). Using the semi-quantitative measurement of *Ψ*m by fluorescence microscope, some scholars have demonstrated that senegenin can maintain the intensity of red fluorescence in a dose-dependent manner. In addition, as the final executor, caspase-3 can cause cell apoptosis. Studies have found that H/R can increase the activity of casepase-3, and senegenin can effectively reverse the changes caused by H/R in a dose-dependent relationship. Besides, experiments have shown that the intracellular Ca^2+^ level of the H/R group was significantly increased, and senegenin can significantly reduce the intracellular Ca^2+^ level and the activity of NADPH oxidase to inhibit H/R injury programmed cell death (Zhu et al., 2016).

In summary, the anti-H/R effect of senegenin is mainly to prevent the mass production of ROS by inhibiting the level of Ca^2+^ and NADPH oxidase, thereby inhibiting intracellular calcium overload, *Ψ*m loss, caspase3 activation and other adverse effects, reducing the cell apoptosis.

#### 2.1.4 Antidepressant Effects

Clinical studies demonstrate that antidepressants have certain side effects. Therefore, it is essential to find new candidates to treat depression. In animal studies, some scholars found that the concentration of central inflammatory cytokines is positively correlated with depression (Young et al., 2014). And there is evidence that activating nuclear transcription factor (NF-κB) and NOD-like receptor protein 3 (NLRP3) inflammasomes can jointly regulate the inflammatory process.

Senegenin has a certain antidepressant effect on the behavioral changes and inflammatory response of mice induced by chronic unpredictable mild stress (CUMS), which regulates the inflammatory pathway of NLRP3 by inhibiting NF-κB, up-regulating the levels of neurotrophic factor proteins (BDNF, NT-3), down-regulating the activity of NLRP3 inflammasomes related proteins, and inhibiting the secretion of IL-1β and TNF-*α*(Chao et al., 2006; Li et al., 2017). It is suggested that senegenin could exert a certain antidepressant effect by inhibiting the release of pro-inflammatory cytokines via NF-κB and NLRP3 signaling pathways.

#### 2.1.5 Anti-Optic Nerve Injury

Optic nerve injury is a common disease in ophthalmology and neurology, and it is also one of the main causes of blindness. Increasing studies have confirmed that axonal injury can lead to oxidative stress occurs in a variety of nerve cells such as retinal ganglion cells (RGCs) (Geiger et al., 2002), and cause apoptosis by affecting the function of mitochondria (Lieven et al., 2003). Therefore, blocking the occurrence of apoptosis in time is the key to protecting nerve cells.

Senegenin was recorded to display good protective effect on H_2_O_2_-induced RGCs damaged at the concentrations of 10, 20 and 40 μM, and such protective effect was most obvious at 40 μM. The mechanism was proved to be associated with its ability to increase the expression of Bcl-2 protein and decrease the expression of Cyt c (Bie et al., 2012). These studies demonstrated that senegenin has the potential to protect the optic nerve and provide new ideas for future clinical medications.

#### 2.1.6 Protective Effects of Nerve Cells in Spinal Cord

Direct or indirect physical trauma to the spinal cord can lead to hemorrhage, edema, neuronal apoptosis, and nerve conduction disruption (Zhang et al., 2015). Improving neuronal survival and promoting axon growth is the key to restore the function of spinal cord injury (SCI) (Agrawal et al., 2010). Regulating Bcl-2/Bax can reduce the apoptosis of spinal cord cells and promote cell survival.

Studies have shown that senegenin significantly improves the motor function of rats with spinal cord contusion, reduces the number of apoptotic cells, and thus exerts a neuroprotective effect. One experiment established a rat spinal cord contusion model, senegenin (30 mg/g) was injected into the tail vein 3 h after injury for three consecutive days. Senegenin significantly reduces the size of syringomyelia and the number of apoptotic cells in the spinal cord. Experiments have found that the mechanism of action is due to the fact that senegenin reduces Bax and Caspase-3, up-regulates Bcl-2, increases the density of nerve fibers in the proximal spinal cord of the rat brain. Additionally, senegenin improved the electrophysiological properties of the hind limbs of rats, as well as promoting the recovery of function after spinal cord contusion (Zhang et al., 2016). Therefore, senegenin may have great therapeutic potential in the treatment of SCI.

### 2.2 Other Pharmacological Effects

Senegenin can delay the degradation/resynthesis of IκBα caused by RANKL, and significantly inhibit the activity of NF-κB, the nuclear translocation and activation of NF-κB p65 subunit (Iotsova et al., 1997), thereby inhibit the effective transcription of genes related to osteoclastogenesis. Prevent excessive osteoclastic activity resulting in osteoporosis and osteolysis (Teitelbaum, 2000). Human osteoarthritis chondrocytes can be inhibited by senegenin by inhibiting PI3K/AKT/NF-κB signaling pathway, inhibiting the expression of MMP-1, MMP-3, and MMP-13 induced by IL-1β, and the production of NO and PGE2 was reduced in human osteoarthritis chondrocytes.

Senegenin also can prevent fulminant liver failure by significantly up-regulating the expression of Nrf2 and HO-1 in a dose-dependent manner (Wilson et al., 2011). Senegenin has a protective effect on LPS/GalN-induced hepatic injury by inhibiting the production of inflammatory cytokines MDA and MPO, inhibiting ASK1, MAPKs and NF-κB.

Senegenin can significantly reduce blood urea nitrogen (BUN) and serum creatinine (Scr), which are important signs of kidney damage (Clarkson et al., 2006), by inhibiting the TLR4/NF-κB signaling pathway. And senegenin can inhibit the Acute kidney injury (AKI) caused by LPS-induced inflammatory response in a dose-dependent manner (Fu et al., 2016; Xu et al., 2017).

Studies have shown that senegenin can significantly inhibit the degradation of IκB, the activation of ERK and p38 MAPK signaling pathways, production of TNF-*α*, IL-1β and IL-6, as well as the expression of COX-2 protein both *in vivo* and *in vitro* (Lv et al., 2015). The treatment of senegenin can not only significantly improve LPS-stimulated pulmonary edema, coagulation and inflammation, but also reduce myeloperoxidase (MPO) activity and lung wet-to-dry weight ratio caused by Acute Lung Injury (ALI). Moreover, senegenin significantly attenuated *Staphylococcus aureus*-induced lung histopathological changes by inhibiting NF-κB activation. Senegenin may be a potential drug for the treatment of pneumonia caused by *Staphylococcus aureus* (Yu et al., 2017).

## 3 Toxicity of Senegenin

Studies have shown that raw Polygalae Radix is irritating. Long-term and high-dose administration of Polygalae Radix can cause gastric mucosal damage and intestinal flatulence, thinning or necrosis (Wang et al., 2018), and the toxicity mainly comes from Polygalae Radix saponins (Guan et al., 2012). However, Wen et al. (2015) proved that senegenin has almost no gastrointestinal toxicity, indicating that senegenin is very valuable for future clinical medications.

An experiment found that rats were still in good condition after oral administration of 500 mg/(kg/d) hydrolyzed saponins by alkali without any toxic symptoms, indicating that the toxicity of senegenin was significantly reduced (Wang et al., 2010). In the isolated intestinal motility test, it was found that Polygalae Radix saponins can cause irregular and strong contraction of the isolated intestine, while senegenin exhibited no significant influences on increasing of intestinal tension (IIT), changing ratio of intestinal tension (CRIT) and amplitude comparing to the control (Wen et al., 2015). This proves that senegenin is almost non-irritating to the gastrointestinal tract. In addition, It is suggested that glycosyl may be highly correlated with gastrointestinal irritation (L. Wen et al., 2014). PGE2 is a protective agent for the gastric mucosa. Senegenin without glycosyl has a weaker effect on reducing PGE2 levels in the stomach, which prevents the loss of gastric mucosal protective function and reduces gastric damage comparing to Polygalae Radix saponins.

Senegenin displays nearly no gastrointestinal or hemolytic toxicity. Considering this, senegenin may be appropriate for clinical use, but the comprehensive systemic toxicology data of senegenin still needs further verification.

## 4 Conclusion

**FIGURE 3:**
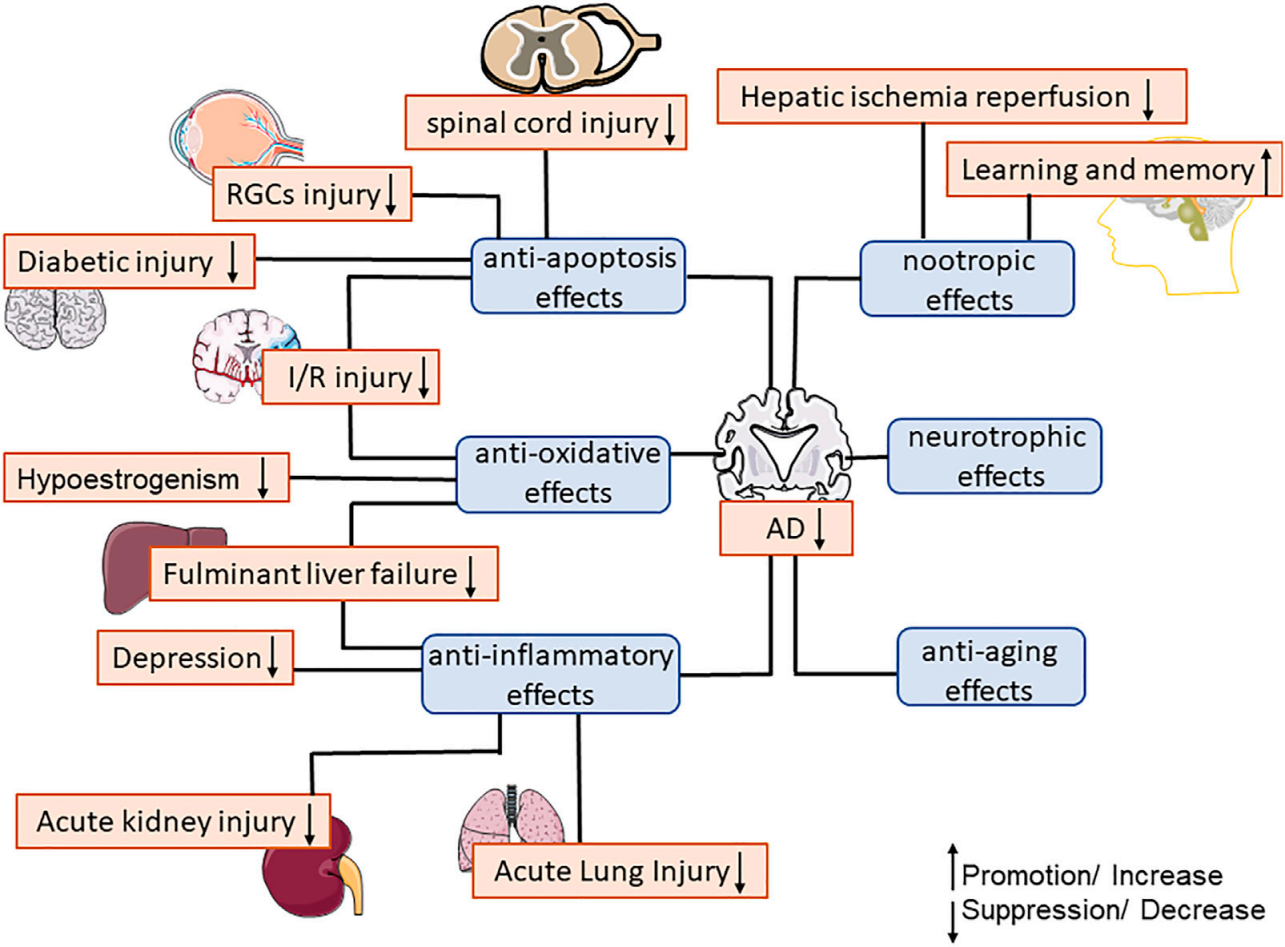
The main pharmacological activities of senegenin.

Polygalae Radix has been widely used for more than 2,000 years as a drug to promote intellectual development in traditional Chinese medicine (TCM). As the main ingredient in Polygalae Radix, senegenin has been proved to have various pharmacological activities such as anti-inflammation (Li et al., 2017), anti-oxidation (Ballard et al., 2011), anti-apoptosis (Zhang et al., 2016), and enhancing cognitive function (Huang et al., 2013). This article summarized the current pharmacological properties and mechanism of senegenin, and explained that the lack of glycosyls of senegenin can significantly reduce gastric mucosal damage, enhance the protection of cognitive function (Wang et al., 2010; Wen et al., 2015). The pharmacological activity of senegenin is shown in Figure 3.

At present, the pathogenesis of neurological diseases focuses on the following theories: The cholinergic hypothesis, that AD is mainly caused by the decrease in the synthesis of the neurotransmitter acetylcholine (Babic, 1999), senegenin can inhibit the activity of AChE, improve the antioxidant capacity, and enhance the cognitive function of learning (Huang et al., 2013). The amyloid hypothesis, that extracellular Aβ deposition is the root cause of AD, and senegenin can inhibit Aβ aggregation through UPP(Hardy and Allsop, 1991). The tau hypothesis, abnormal tau protein initiates the disease cascade (Mudher and Lovestone, 2002), and senegenin can inhibit the abnormal phosphorylation of tau by regulating GSK-3β/CDK-5 (Chen Y. J. et al., 2012). In addition, senegenin can reduce neuroinflammatory response by regulating NLRP3/Nrf2/HO-1, and regulate PI3K/Akt signaling pathway to nourish nerve cells, regulate the expression of NR2B/HGN protein, play a protective effect on learning and memory impairment (Pi et al., 2013; Luo et al., 2016; Lv et al., 2016; Lin et al., 2018). At the same time, Senegenin in the treatment of postoperative and cognitive dysfunction, as well as ischemia-reperfusion injuries is associated with the regulation of JNK signaling pathway, the inhibition of NADPH oxidase and caspase-3 activity, the increase of Bcl-2/Bax (Zhang et al., 2016).

## 5 Further Perspective

A significant neuroprotective effect of senegenin is its capability to clear the abnormal deposition of Aβ, inhibit the abnormal phosphorylation of tau, reduce oxidative stress, inhibit cell apoptosis, enhance synaptic plasticity and learning and memory ability. Senegenin is a potential drug for AD. Although clinical studies have found that in the treatment of moderate to severe AD, the effect of adding senegenin was significantly enhanced (Chang and Teng, 2018; Dong and et al., 2018), there are too few clinical studies. In order to speed up the clinical application of senegenin, there are several key issues that need to be solved:

Firstly, the current literature has not made a comprehensive evaluation of the systemic toxicology of senegenin, and its acute toxicity and long-term toxicity data still need to be further verified. This will help researchers determine the safe dosage and range of medications, provide a basis for the structural transformation of new drugs, and ensure the safety of clinical medications.

Secondly, the extraction and processing of TCM directly affect the safety and effectiveness. However, the current technology for separating and extracting senegenin is still traditional and lacks objective technical parameters. The efficacy of Polygalae Radix may change during processing, there is an urgent need to find a standardized extraction and purification method for senegenin, to achieve its standardized production, to establish safe, stable, reliable and specific quality guidelines. Furthermore, a new method must be developed to evaluate the relationship between the effectiveness and toxicity of senegenin, and to achieve a balance to maximize its clinical effects.

Finally, previous studies have reported that after intragastric administration, the kinetics of senegenin in rats manifests as the non-compartmental model. The T_max_ vale of senegenin was recorded to be 0.75 h, indicating that senegenin can be absorbed rapidly; the T_1/2_ value was 12.66 ± 5.29 h, which showed that the metabolism of senegenin is slow (Lin et al., 2014). It proves that senegenin has the potential to become a clinical drug. According to a recent pharmacokinetic study, the T_1/2_ value of senegenin was 2.6 ± 0.6 h, which was significantly different from previous studies (Shen et al., 2022). Although the scholars explained that the experimental animals and mixed drugs of the two studies were different, it also proved that the research on senegenin was not sufficient, and the pharmacokinetic study of senegenin in humans needs further exploration. Senegenin may be considered for sustained- or controlled-release administration if it is rapidly metabolized. Future research should use a variety of animal models and multiple methods of administration to study its pharmacokinetics. Similarly, scholars can also use the methods of Serum pharmacochemistry of TCM and Chinmedomics to explain the biological nature of syndromes in order to understand the body’s effects on senegenin. In addition, establish a pharmacokinetic-pharmacodynamic model that conforms to the characteristics of TCM, study the relationship between senegenin “dose time-drug concentration-drug effect,” clarify the material basis for the pharmacodynamics of senegenin and guide rational clinical use.
